# Inflammation—the role of TRPA1 channel

**DOI:** 10.3389/fphys.2023.1093925

**Published:** 2023-02-16

**Authors:** Kaifang Yao, Baomin Dou, Yue Zhang, Zhihan Chen, Yanwei Li, Zezhi Fan, Yajing Ma, Simin Du, Jiangshan Wang, Zhifang Xu, Yangyang Liu, Xiaowei Lin, Shenjun Wang, Yi Guo

**Affiliations:** ^1^ Research Center of Experimental Acupuncture Science, Tianjin University of Traditional Chinese Medicine, Tianjin, China; ^2^ School of Acupuncture & Moxibustion and Tuina, Tianjin University of Traditional Chinese Medicine, Tianjin, China; ^3^ National Clinical Research Center for Chinese Medicine Acupuncture and Moxibustion, Tianjin, China; ^4^ School of Chinese Medicine, Tianjin University of Traditional Chinese Medicine, Tianjin, China

**Keywords:** TRPA1, antagonist, agonist, ion channels, inflammatory factors, immune cells, inflammatory diseases

## Abstract

Recently, increasing numbers of studies have demonstrated that transient receptor potential ankyrin 1 (TRPA1) can be used as a potential target for the treatment of inflammatory diseases. TRPA1 is expressed in both neuronal and non-neuronal cells and is involved in diverse physiological activities, such as stabilizing of cell membrane potential, maintaining cellular humoral balance, and regulating intercellular signal transduction. TRPA1 is a multi-modal cell membrane receptor that can sense different stimuli, and generate action potential signals after activation *via* osmotic pressure, temperature, and inflammatory factors. In this study, we introduced the latest research progress on TRPA1 in inflammatory diseases from three different aspects. First, the inflammatory factors released after inflammation interacts with TRPA1 to promote inflammatory response; second, TRPA1 regulates the function of immune cells such as macrophages and T cells, In addition, it has anti-inflammatory and antioxidant effects in some inflammatory diseases. Third, we have summarized the application of antagonists and agonists targeting TRPA1 in the treatment of some inflammatory diseases.

## 1 Introduction

The transient receptor potential (TRP) protein family is a class of non-selective gated cation channels located on the cell membrane and composed of several members. Based on the homology of their amino acid sequences, mammalian TRP channels can be classified into the following seven subfamilies: TRPC (Canonical), TRPV (vanilla loid), TRPM (Melastatin), TRPP (polycytin), TRPML (Mucolipin), TRPA (Ankyrin), and TRPN (NOMP-C) (Talavera et al., 2020). TRPA1 is a member of the TRP family. Its gene was cloned from lung fibroblasts in the year 1999. Owing to the presence of multiple ankyrin repeats at the N-terminal, and its gene name is also ANKTM1 (Jaquemar et al., 1999). TRPA1 usually functions as a homotetramer with six transmembrane domains (S1–6); S1 and S2 form an extracellular loop structure and contain 18 ankyrin repeat domains at its N-terminus, which may be involved in inter-protein interactions, such as binding with phospholipase C (PLC) and calcium (Ca^2+^) (Peng et al., 2015). TRPA1 is composed of approximately 1,100 amino acids and has a molecular weight of 120–130 kDa. It can sense a variety of physical and chemical stimuli and participate in important life activities, such as body temperature, taste, hearing, and osmotic pressure (Logashina et al., 2019).

TRPA1 is abundantly expressed, mainly in a subset of primary sensory neurons, as well as in non-neuronal cells such as macrophages, dendritic cells, T-lymphocytes, neutrophils, and mast cells (Jain et al., 2011; Mukhopadhyay et al., 2011; Oh et al., 2013; Naert et al., 2021). TRPA1 can be activated *via* exogenous stimuli in three different ways: exogenous stimuli activate protein kinase C (PKC) through G protein-coupled receptors (GPCRs) and receptor tyrosine kinases (RTKs) to regulate the activity of the TRPA1 channels; Low-molecular-weight organic matter and endogenous esters act as ligands of TRPA1 to activate the channel, Moreover, temperature changes and mechanical stimulation directly act on the TRPA1 channel to promote its opening and activation (Logashina et al., 2019). Several studies have demonstrated that TRPA1 is involved in various physiological and pathological processes of the body, and it is considered a sentinel molecule of cellular stress, tissue damage, and inflammatory responses, as well as an important mediator involved in immune responses to inflammatory diseases (Straub, 2014; Viana, 2016). Garrison et al. (Garrison and Stucky, 2011) proposed that TRPA1 is both a booster of inflammatory responses and a detector of inflammatory mediators. TRPA1 can be activated directly or indirectly by inflammatory signaling molecules or by heat and oxidants in the inflammatory microenvironment; moreover, it is involved in the activation of transcription factors (TF) and protein kinases (PK) in some pro-inflammatory or anti-inflammatory signaling cascades. Meanwhile, TRPA1 has been reported to be related to neurogenic inflammation, osteoarthritis, allergic dermatitis, asthma, inflammatory bowel disease, migraine, cancer pain, gout, and other diseases, and it is a promising drug therapy target (Moilanen et al., 2012; Lucaciu and Connell, 2013; Nassini et al., 2014; Zygmunt and Högestätt, 2014; Abdel-Magid, 2021). In this paper, we reviewed the mechanism through which immune cell activation and inflammatory factor release regulates oxidative stress and participates in the body’s inflammatory response and inflammatory diseases *via* the activation and inhibition of the TRPA1 channels was reviewed, and the application of TRPA1 antagonists and agonists in disease treatment was summarized to provide a scientific basis for the prevention and treatment of inflammatory diseases with potential therapeutic targets.

## 2 Pro-inflammatory roles

### 2.1 Association of TRPA1 activation with pro-inflammatory responses

The onset of inflammation leads to immune cell activation, which causes the release of different inflammatory mediators that interact with the TRPA1 channels. Inflammatory factors cause activation or sensitization of the TRPA1 channel located at the end of pain receptors, increase the influx of Ca^2+^, cause neurogenic inflammatory response and release of neuropeptides, such as substance P (SP), neurokinin A (NKA), and calcitonin dene-related peptide (CGRP), thereby initiating a series of signal-transduction pathways to regulate the inflammatory process (Dai et al., 2007; Kunkler et al., 2011; Moilanen et al., 2015a; Nummenmaa et al., 2016). TRPA1 contributes to the transition of inflammatory and immune responses from an early defense to a chronic pathological state. TRPA1 coordinates a series of cellular processes such as cytokine production through indirect regulation of the intracellular pathways (Landini et al., 2022). The inflammatory factors mediating TRPA1 activation and pro-inflammatory response include interleukin-1 (IL-1), tumor necrosis factor-α (TNF-α), interleukin-6 (IL-6), and interleukin-8 (IL-8) (Bautista et al., 2013; Lin et al., 2015; Feng et al., 2017; Kanda et al., 2018).

#### 2.1.1 Inflammatory factors

##### 2.1.1.1 IL-1 and TRPA1

The interleukin-1 family is an important cytokine that plays a key role in regulating the body’s immune response and inflammatory responses. Presently, the interleukin-1 family mainly includes 11 cytokines (IL-1α, IL-1β, IL-1RA, IL-18, IL-36A, IL-37, and IL-33), which play an important role in the pathophysiology of inflammation (Yazdi and Ghoreschi, 2016). Past studies have reported the interaction between TRPA1 and IL-1 family during the process of inflammation and recorded increased expression of TRPA1, IL-1β, and IL-18 in the lung tissues after cigarette smoking. The experiment was performed using cigarette smoke extract (CSE) to induce asthma and particulate matter (PM2.5) for the preparation of an asthma exacerbation model. The inhibition of TRPA1 might also reduce the IL-1β and IL-18 expression (Wang et al., 2019). Xu et al. (2019) proved that the inhibition of only TRPA1 or the combined inhibition of TRPA1 and transient receptor potential (TRP) vanilloid 1 (TRPV1) channels cause the inhibition of lung injury NOD-like receptor thermal protein domain-associated protein 3/cysteinyl aspartate specific proteinase (NLRP3/caspase-1) pathway, thereby reducing the expression of IL-1β. Monosodium urate (MSU) induces IL-1β release in the synovial tissue in a model of acute gout, Which then stimulates plasma protein extravasation and TRPA1 production. TRPA1 antagonist treatment significantly reduces murine IL-1β, thereby reducing hyperalgesia and edema (Trevisan et al., 2014). Sulfur mustard (SM) and 2-chloroethyl ethyl sulfide (CEES)-induced mouse ear bubble model activates TRPA1 in skin sensory nerves, releasing CGRP and pro-inflammatory factors IL-1β, C-X-C ligand (CXCL1), and matrix metalloprotein 9 (MMP-9). The use of TRPA1 inhibitor HC-030031 can reduce the expression of IL-1β (Achanta et al., 2018). After the activation of TRPA1 on different cell membranes, it mediates the release of cellular inflammatory factors. Past studies have reported the activation of TRPA1 in scalp skin keratinocytes from face-lift surgery patients to promote the release of IL-1α, IL-1β, and prostaglandin (PGE2) (Atoyan et al., 2009). Doxorubicin (Dox) provokes a series of inflammatory responses and causes the deterioration of myocardial functions, in a mouse model of Dox-induced cardiac injury, the activation of TRPA1 in cardiomyocytes increased IL-1β secretion and endoplasmic reticulum stress, thereby resulting in cardiomyocyte apoptosis (Wang S. et al., 2018). Hypoxia-induced activation of TRPA1 in human glioblastoma increases the expression of IL-1β, IL-18, caspase 3, and caspase 9. It promotes mitochondrial oxidative stress and tumor cell apoptosis (Deveci et al., 2019). Inflammatory factors play an important role in endometriosis, which leads to local vasoconstriction and induces endometrioid-related pain, TRPA1 antagonists inhibit the levels of IL-1β, IL-6, TNF-α, and PGE2 in mouse serum, thereby exerting an anti-inflammatory effect (Zhu et al., 2022). IL-33 is highly expressed in barrier sites, and acting *via* the suppression of tumorigenicity two receptor (ST2). It triggers neutrophil-dependent reactive oxygen species (ROS) production through ST2, which, in turn, activates dorsal root ganglion (DRG) neurons. Ultimately, the TRPA1 channels produce pain sensations. Thus, the IL-33/ST2 axis involved in the immune response may prove as a promising target or new therapeutic approach to improve gout (Yin et al., 2020). Amyloid-β (Aβ) activates TRPA1 in astrocytes and microglia to trigger chronic inflammation, thereby releasing cytokines, pro-inflammatory mediators, and ROS. This event leads to the progression of Alzheimer’s disease (AD). Therefore, the inhibition of the TRPA1 channel reduces the expression of pro-inflammatory cytokines IL-1β and IL-6 as confirmed in AD mice through the nuclear factor kappa-B (NF-kB) signaling pathway (Lee et al., 2016). These studies prove that the activation of TRPA1 mediates the inflammatory response, thereby releasing some IL-1 family inflammatory factors.

##### 2.1.1.2 TNF-α and TRPA1

TNF-α is involved in initiating inflammatory responses. In the early stages of inflammatory responses, TNF-α stimulates endothelial cells and increases the release of vascular adhesion factors, IL-1β, and IL-6. Simultaneously, it also induces neutrophils to accumulate at the inflammation site, thereby aggravating the inflammatory response. Past reports have suggested that TNF-α exacerbates inflammatory responses by activating the target cells of NF-κB and c-Jun N-terminal kinase (JNK) signaling pathway (Aggarwal et al., 2012). When keratinocytes were stimulated with TNF, the inflammatory signaling pathways NF-κB, JNK, and mitogen-activated protein kinases (p38 MAPK) were activated and the expression of TRPA1 increased in a time- and dose-dependent manner. Furthermore, the inhibition of TRPA1 reduced monocyte chemotactic protein-1 (MCP-1) in mouse keratinocyte synthesis (Luostarinen et al., 2021). As also reported elsewhere, TRPA1 inhibitor prevented the induction of CD25 and CD69 and the secretion of cytokines TNF-α and interferon-gamma (IFN-γ) in ConcanavalinA (ConA) or by T-cell receptor (TCR)-stimulated T cells (Sahoo et al., 2019). TRPA1 is also important in TNF-α mediated visceral pain. The TNF-α level in the peripheral blood mononuclear cell supernatants of patients with inflammatory bowel disease (IBD) is elevated, and the presence of TRPA1 antagonists abolishes the inflammatory effects of TNF-α (Campaniello et al., 2017). TNF-α mediates the joint inflammation in rheumatoid arthritis (RA). The progression of this disease is dependent on the expression of TRPA1 in peripheral blood leukocytes. The activation of TRPA1 amplifies joint inflammation. Treatment with the anti-rheumatic drug leflunomide (LFN) reduces the expression of TRPA1 and plasma TNF-α in patients (Pereira et al., 2017). Based on past reports, in human fibroblast-like synoviocytes, TNF-α induces *TRPA1* expression through the activation of the NF-κB signaling pathway, downstream transcription factor hypoxia-inducible factor-1 (HIF1α), and increasing the release of the cytokine IL-8 and MMP9 (Hatano et al., 2012; Yap et al., 2020). The TRPA1 expression is elevated in the odontoblasts of carious teeth and tooth bleaching activates TRPA1, which then promotes the absorption of intracellular calcium, increases the secretion of adenosine triphosphate (ATP), and induces inflammatory pain. Simultaneously, TRPA1 upregulates TNF-α and IL-6 (Chen et al., 2021). Hypothermia prolongs the inflammatory response of monocytes, thereby acting as a risk factor for postoperative complications. As reported earlier, TRPA1 is expressed on the surface of monocytes; blocking this expression increases the secretion of protective IL-10 and reduces the secretion of TNF-α (Billeter et al., 2015). Therefore, TRPA1 and TNF-α interact during the inflammatory response. The TNF-α inflammatory mediators upregulate the expression of TRPA1, which then activates TRPA1, eventually resulting in the release of TNF-α.

##### 2.1.1.3 IL-6 and TRPA1

In addition to TNF-α and the IL-1 families, some other inflammatory factors play a central role in regulating the inflammatory response. IL-6—a polypeptide substance—synergizes with TNF-α to induce T-cell proliferation, thereby leading to immune dysfunction (Tanaka et al., 2014). TRPA1 also regulates the synthesis of IL-6 in chondrocytes. The levels of IL-6 were elevated in patients with osteoarthritis (OA). inhibiting TRPA1 reduced the LPS-induced IL-6,IL-1β, TNF-α secretion and relieved the arthritis symptoms (Yin et al., 2018). Similarly, in experimental osteoarthritis, IL-6 was highly expressed in the chondrocytes of wide-type (WT) mice, whereas the expression of IL-6 was significantly downregulated in TRPA1-knockout (KO) mice.This event can be attributed to the expression of different genes toll-like receptor 2 (TLR-2), cluster of differentiation 36 (CD36), and as well as the cytokines IL-33, activation of TLR2 increases IL-6 production in the synovium (Papathanasiou et al., 2011); CD36 regulate IL-6 from dendritic cells (Lim et al., 2014); IL-33 is associated with mast cell IL-6 (Kondo et al., 2021). These genes were expressed at lower levels in TRPA1-KO mouse chondrocytes when compared with that in WT mice (Nummenmaa et al., 2020). N-acylethanolamines anandamide (AEA)—a dual fatty acid amide hydrolase/cyclooxygenase-2 (FAAH/COX-2) inhibitor—inhibited MAPK signaling in patients with RA and desensitizes TRPA1 and downregulates IL in COX-2-dependent MMP-3 levels (Lowin et al., 2015). The oral administration of di-iso-nonyl phthalate (DINP) aggravates allergic dermatitis tissue damage in sensitized mice, and researchers believe that DINP enhances the TRPA1 expression through NF-κB signaling, which increases the levels of IL-6 and T-helper type2 (Th2) cytokines (LaMotte, 2016; Kang et al., 2017). TRPA1 plays a crucial role during atopic dermatitis (AD) pathogenesis in mice. TRPA1-KO mice exhibited reduced expression of the pro-inflammatory factor IL-6 and alleviated pruritus in mice with 2,4-dinitrochlorobenzene (DNCB)-induced experimental AD (Zeng et al., 2021). Neurogenic inflammation induced by LPS is mainly dependent on the activation of TRPA1 in nociceptive sensory neurons. The interaction of LPS with the myeloid differentiation protein-2 (MD2)-TLR4 complex causes the activation of NF-κB and the production of IL-6. Moreover, TRPA1-KO inhibits TLR4 and alleviates LPS-induced inflammatory response in mice (Meseguer et al., 2014), which confirms that the expression of IL-6 is regulated by TRPA1 and that the activation of TRPA1 can regulate the release of IL-6, which in turn mediates the inflammatory response and the subsequent release of inflammatory factors.

##### 2.1.1.4 IL-8 and TRPA1

IL-8 can be chemotactic and activate neutrophils, promote their degranulation, release superoxide and lysosomal enzymes, and aggravate the inflammatory response (Baggiolini and Clark-Lewis, 1992). Past reports suggest that the TRPA1 expression on non-neuronal cells and inflammatory mediators could modulate its function. the upregulation of TRPA1 in human fibroblast cells increases IL-8 release and the MMP9 expression, while the inhibition of TRPA1 reduces the release of IL-8 and the NLRP3/caspase1 inflammasome complex (Yap et al., 2020). LPS causes pain and acute vascular responses by relying on the activation of TRPA1 in pain sensory neurons (Meseguer et al., 2014). LPS activates TRPA1 on lung epithelial cells (LECs), which increases the Ca^2+^ influx, the activation of nicotinamide adenine dinucleotide phosphate (NADPH) oxidase, and LECs intracellular increase of ROS, which in turn activates the MAPK/NF-κB signaling pathway, resulting in an increase in the release of IL-8 (Nie et al., 2016; Ko et al., 2020). The acyl-glucuronide metabolite of ibuprofen inhibited the release of IL-8 in bronchial epithelial cells induced by the TRPA1 agonist AITC, thereby exerting anti-inflammatory and analgesic effects (De Logu et al., 2019). These studies demonstrated that the activation of TRPA1 promotes the inflammatory response by inducing the release of IL-8 through the inflammatory signaling pathways.

### 2.2 Neuropeptides and TRPA1

TRPA1, a pain and thermal stimulus sensor that is expressed at high levels in C fibers associated with neuropathic pain, has been associated with neurogenic inflammation (De Logu et al., 2016; Bamps et al., 2021). Sensory neurons innervating the skin, airway, and gastrointestinal tract sense stimuli and activate TRPA1 on cell membranes, which releases neuropeptides such as NKA, SP, and CGRP, thereby promoting and regulating inflammatory responses (Andersson et al., 2008; Andrè et al., 2008; Bessac et al., 2008; Sasaki et al., 2014). These neuropeptides stimulate mast cells and lymphocytes and produce inflammatory factors, thereby inducing neurogenic inflammation. Moreover, it forms a molecular signal transmission process of exogenous stimuli-TRPA1, which includes the activation Ca^2+^ influx-neuropeptide release-neurogenic inflammation and the molecular signaling process of endogenous stimulation-PLC/PKC-TRPA1-neurogenic inflammation (Benemei et al., 2014). TRPA1 inhibition reduces sensory neuron activation and the neuropeptide-release levels (Clapham et al., 2001). An intraarticular injection of sodium iodoacetate (MIA) in mice mimics the joint pathology of OA. In chondrocytes, MIA induces ROS to activate TRPA1, which results in the release of SP, that acts on neurokinin-1 (NK1) to induce acute inflammatory responses that are alleviated by pretreatment with TRPA1 antagonists inflammatory edema (Moilanen et al., 2015b). Moreover, the activation of TRPA1 triggers an intestinal inflammatory response, releasing SP and CGRP. This event is induced by the TRPA1 agonist trinitrobenzene-sulfonic-acid (TNBS) that promotes the production of IBD-like symptoms in mice (Engel et al., 2011). The topical application of oxazolone and urushiol in a mouse model of allergic contact dermatitis (ACD) activates TRPA1, which enhances the release of neurogenic inflammatory and pruritic mediators such as SP and NKA (Liu et al., 2013). The topical application of cinnamaldehyde on mouse ears causes neutrophil infiltration, and acute inflammatory responses with edema formation, could be dependent on TRPA1 receptor activation. while TRPA1 and NK1 receptor antagonists prevent cinnamaldehyde-induced ear edema (Silva et al., 2011). TRPA1-mediated detrusor contraction involves the stimulation and secretion of sensory afferent neuropeptides and prostaglandins, whose agonists causes bladder contractions and pain-like behaviors accompanied by increased levels of SP and CGRP (Juszczak et al., 2011; Skryma et al., 2011; Weinhold et al., 2018). These reports confirm that the activation of TRPA1 mediates the inflammatory response and the release of neuropeptides, which promotes neurogenic inflammation.

### 2.3 TRPA1/NOX pathway

The TRPA1/NADPH oxidase 1 (TRPA1/NOX1) pathway mediates oxidative stress in peripheral Schwann cells and macrophages and is involved in inflammatory responses. Other inflammatory mediators released by macrophages such as reactive nitrogen species (RNS) and ROS are known to activate TRPA1 (De Logu et al., 2022). The activation of TRPA1 regulates the influx of Ca^2+^ in the mitochondria, inducing the increase of ROS in the mitochondria, leading to mitochondrial dysfunction and thereby induces apoptosis and possibly chronic inflammatory diseases (Nesuashvili et al., 2013). In mice with partial sciatic nerve ligation, neuroinflammation activates Schwann cell TRPA1 to cause NOX1-dependent hydrogen peroxide (H_2_O_2_) release, and silencing of Schwann cell NOX1 reduces nerve injury-induced macrophage infiltration and ectopic pain (De Logu et al., 2017). TRPA1 (mRNA and protein) expressed in retinal cells mediates retinal inflammation *via* the TRPA1/NOX1 pathway. In WT mice, the retinal cell numbers and thicknesses were significantly different on days 2 and 7 after ischemia and reperfusion (I/R) reduction; the TRPA1-KO mice attenuated the number of infiltrating macrophages and the level of caspase-3 (Souza Monteiro de Araújo et al., 2020). The TRPA1/NOX pathway in trigeminal ganglion (TG) neurons is also involved in triglyceride-induced migraine, and ectopic pain is attenuated in TRPA1-KO mice or the selective deletion of TRPA1 in sensory neurons (Marone et al., 2018). In the complex regional pain syndrome (CRPS-I) mouse model, macrophages and Schwann cell TRPA1 maintain chronic neuroinflammation. In mice with the selective deletion of Schwann cell TRPA1, the oxidative stress marker 4-hydroxynonenal (4-HNE) and pain are weakened (De Logu et al., 2020). Thermal injury promotes tissue inflammation, which is involved in the inflammatory response of thermal lesions, resulting in the augmented production of reactive oxygen species, leading to the activation of TRPA1. In addition, the TRPA1 antagonist HC-030031 reduces edema and neutrophil infiltration as well as the levels of H_2_O_2_ and NADPH oxidase activity after heat injury in mice (David Antoniazzi et al., 2018). These reports prove that the activation of TRPA1/NOX pathway mediates the inflammatory responses.

## 3 Therapeutic applications of TPRA1 antagonists in inflammatory diseases

### 3.1 Pain

TRPA1 is an important mediator of pain (Tsagareli and Nozadze, 2020). In the peripheral tissues, a variety of harmful inflammatory stimuli are detected in sensory nerve endings. These stimuli activate TRPA1 to cause neurogenic inflammation, transmit pain information to the primary sensory center of the spinal cord, and activate neurons to release SP and CGRP to transmit pain (Cattaruzza et al., 2010). TRPA1 has become a new target for the treatment of inflammatory pain, neuropathic pain, and cancer pain.

In inflammatory pain, blocking the TRPA1 receptor facilitates reduction of chronic inflammation and pain associated with the complete Freund’s adjuvant (CFA) arthritis (Fernandes et al., 2011; Horváth et al., 2016). The TRPA1 antagonist HC-030031 and the COX inhibitor ibuprofen inhibited the plantar edema induced by *C. japonica* in mice (Moilanen et al., 2012). Pain-related behaviors induced by intraplantar injection of Cd were found to be significantly attenuated in TRPA1-KO mice (Miura et al., 2013). Pretreatment with the TRPA1 antagonist HC-030031 significantly inhibited xylene-induced nociceptive responses (Norões et al., 2019). An intraplantar injection of histamine lead to significant thermal hyperalgesia, and pretreatment with the TRPA1 antagonist HC-030031 significantly reduced thermal hyperalgesia and mechanical allodynia elicited by chloroquine (Tsagareli et al., 2020). The TRPA1 antagonist A-967079 prevents the conversion of acute pancreatitis (AP) to chronic pancreatitis (CP) and intervenes in early pancreatitis (Schwartz et al., 2013). PERK-eIF2a ATF4-CHOP axles play an important role in endoplasmic reticulum stress and induces apoptosis by upregulating CCAAT/enhancer-binding protein homologous protein (CHOP) and other apoptosis-related factors. TRPA1 inhibitor inhibits endoplasmic reticulum stress by downregulating the PERK/eIF2α/ATF-4/CHOP pathway and reducing oxidative stress and apoptosis in periodontitis mice, thereby improving periodontitis (Morii et al., 2020; Liu et al., 2022). Cyclophosphamide has long been applied for the treatment of lymphom. However, the urologica l disturbances present the major limiting factor in its use, while the TRPA1 receptor was identified in neuronal and non-neuronal structures of the lower urinary tract. Moreover, the systemic application of TRPA1 antagonist HC-030031 in rats effectively reduces cyclophosphamide-induced bladder mucosal injury (Meotti et al., 2013).

TRPA1 is closely related to migraine attacks (Benemei et al., 2013). Inflammatory mediators activate TRPA1 on neurons and glial cells. Demartini et al. (2022) confirmed TRPA1 antagonism prevents multiple changes in the inflammatory pathways by modulating glial activation. TRPA1 antagonist ADM-12 relieves acute and chronic migraine in rats induced by nitroglycerin, reduces the activation of microglia and astrocytes in the caudal region of the trigeminal nucleus, and decreases the expression of pro-inflammatory factors in the medulla oblongata and trigeminal ganglion. In an infraorbital nerve model of trigeminal pain, TRPA1 activation increases oxidative stress, thereby releasing byproducts from aggregated monocytes at the site of nerve injury, while the perineural administration of HC-030031 reduces pain (Trevisan et al., 2016) Trigeminal TRPA1 is activated by exposure to environmental stimuli, and dual TRPV4/TRPA1 inhibitors have been proven to be effective in suppressing trigeminal neuralgia by reduced release of CGRP (Edelmayer et al., 2012; Benemei et al., 2013). Activated protein kinase (AMPK) is an intracellular energy sensor that monitors and regulates energy expenditure. Its activation reduces the amount of membrane-associated TRPA1, inhibits the TRPA1 activity in DRG neurons, and alleviates diabetic neuropathic pain (Wang Z. et al., 2018). Methylglyoxal (MGO) directly activates TRPA1 through its intracellular binding site, which leads to the pathogenesis of diabetic neuropathy, On the other hand, an intrathecal injection of TRPA1 blockers reduces itching and pain in diabetic mice (Eberhardt et al., 2012; Huang et al., 2016; Cheng et al., 2019).

Cancer pain is a mixed pain that includes both inflammatory and neuropathic pains. Current studies suggest that the peripheral mechanism of cancer pain is mainly related to local inflammation and that the activation of peripheral ion channels macrophages colony-stimulating factor (M-CSF) is upregulated in the sciatic nerve trunk, which expands macrophages and activates TRPA1 (Zajączkowska et al., 2019). In a cancer pain model, the M-CSF/macrophages/Schwann cell TRPA1 pathway functions to maintain pain and target the deletion of TRPA1 to reduce pain (De Logu et al., 2021). The triterpenoids of riterpenoids act as antagonists of TRPA1, exerting anti-inflammatory effects to reduce cancer pain (Mäki-Opas et al., 2019). Repeated administration of HC-030031 has an antinociceptive effect on the pain of metastatic bone cancer in mice (de Almeida et al., 2021). HC-030031 or A967079 inhibitors attenuated mechanical/cold hypersensitivity in a mouse hind paw B16-F10 melanoma cell-induced cancer pain model (Antoniazzi et al., 2019).

### 3.2 Asthma

The expression of TRPA1 in CD4^+^ T cells of different asthmatic mice suggests the possible involvement in the neuroimmune interaction of airway inflammation in asthmatic mice (Li et al., 2020). GDC-0334 is a potent oral TRPA1 antagonist that inhibits the effects of TRPA1 on airway smooth muscles and sensory neurons, reduces edema, dermal blood flow, cough, and allergic airway inflammation, and treats asthma (Balestrini et al., 2021).

### 3.3 Central nervous system diseases

TRPA1 is expressed in astrocytes and oligodendrocytes in the central nervous system (Shigetomi et al., 2011; Shigetomi et al., 2013). The inhibition of TRPA1 may be effective in the treatment of multiple sclerosis, AD, and depression. TRPA1 is a potential target for neuroprotection (Shigetomi et al., 2013). The activation of TRPA1 may regulate the MAPK pathway, the transcription factor c-Jun, and the pro-apoptotic B-cell lymphoma-2 (Bcl-2) family, leading to enhanced apoptosis in oligodendrocytes, while its ablation reduces apoptosis in mature oligodendrocytes, thereby significantly reducing demyelination (Bölcskei et al., 2018). Glutamate release and the activation of oligodendrocyte N-methyl-D-aspartate (NMDA) receptors are known pathological basis of hypoxia-induced white matter damage. Blocking oligodendrocyte TRPA1 reduces post-stroke energy deprivation and reduces myelin damage during ischemia (Hamilton et al., 2016). TRPA1 has been linked to amyloid production and astrocyte hyperactivity in neurodegenerative diseases. TRPA1-Ca^2+^-PP2B-NF-κB signaling plays a key role in regulating AD inflammation and pathogenesis. Aβ triggers TRPA1 to influx Ca^2+^ and then increases protein phosphatase 2B (PP2B) activity, which then activates NF-κB and induces astrocytes to produce pro-inflammatory cytokines, thereby accelerating the development of AD. Antagonizing TRPA1 channels inhibits the activation of PP2B and the activity of astrocytes and NF-κB (Lee et al., 2016). TRPA1-KO mice attenuated Aß1-induced neurotoxicity and memory loss in the mouse basal ganglia forebrain (Payrits et al., 2020). The interaction between astrocytes and neurons in the progression of AD has been recently studied. The intraperitoneal injection of the inhibitor HC-030031 in the AD mouse model has been reported to restore the activity of astrocytes in the hippocampus and maintain the integrity of the neuron structure, thereby preventing irreversible damage to neuronal functions (Paumier et al., 2022). Moreover, it has been reported that TRPA1 in the central nervous system affects the neural activity by activating Ca^2+^ signals in astrocytes, which regulates the depressive behavior process in AD. The use of the antagonist HC-030031 in mouse hippocampal CA1 astrocytes abolished Ca^2+^ hyperactivity in astrocytes (Bosson et al., 2017). Therefore, TRPA1 participates in central nervous system diseases by regulating neural circuits and intracellular calcium homeostasis.

In conclusion, TRPA1 plays an pro-inflammatory role in inflammatory diseases by the release of IL-1, TNF-α, and IL-6 while CGRP promotes the inflammatory response and local tissue damage and mediates the occurrence and development of inflammation. Antagonistic TRPA1 is mainly involved in treating pain, asthma, pneumonia, and other diseases caused by neurogenic inflammation.

## 4 Anti-inflammatory roles.

### 4.1 Association of TRPA1 activation with anti-inflammatory responses

The pro-inflammatory role of TRPA1 has been widely confirmed, albeit recent studies suggest that TRPA1 plays an anti-inflammatory, antioxidant, and anti-inflammatory roles in certain inflammatory diseases such as cardiovascular diseases, psoriasis, enteritis, central nervous system diseases, and renal injury (Kun et al., 2014). The existing literature depicts the anti-inflammatory effect of TRPA1 on the regulation of the growth, activation control, and metabolism of immune cells such as T cells and macrophages, thereby confirming the importance of TRPA1 in the regulation of immune cells (Wu et al., 2021).

#### 4.1.1 Inflammatory cells and TRPA1

##### 4.1.1.1 T cells

The activation of T cells is a key part of the immune response, and TRPA1 plays an important role in the T cell activation. In the imiquimod (IMQ)-induced psoriasis model, IMQ acts as an agonist of TRPA1 and activates CD4^+^ T cells through TRPA1, which in turn reduces the release of neuropeptides from the nerve endings, attenuates dendritic cell activation, reduces the release of IL-12 and IL-23, and reduces T helper type l (Th1) or T helper typel7 (Th17) T cells and skin inflammation (Kemény et al., 2018; Wei et al., 2020). TRPA1 is associated with the self-defense system. The activation of TRPA1 promotes oxidative stress tolerance in tumor cells (Takahashi et al., 2018). It has been reported that TRPA1 is involved in the pathogenesis of cancer and other inflammatory diseases, possibly by regulating the metabolism of CD8^+^ T cells. TRPA1 deficiency in CD8+T cells under CD3/CD28 co-stimulation conditions results in increased respiratory responses and glycolysis (Forni et al., 2021). TRPA1 is protective in a T-cell-mediated colitis model by inhibiting the TRPV1 activity in CD4^+^ T cells (Bertin and Raz, 2016). The deletion of TRPA1 in mouse CD4^+^ T-cells upregulates proinflammatory cytokines TNF-α, IFN-γ, and IL-2. The expression of Th1 cells increases the proportion of Th1 cells, thereby aggravating intestinal inflammation (Bertin et al., 2017). In conclusion, TRPA1 participates in the activation process of T cells related to inflammatory diseases and exerts an anti-inflammatory effect.

##### 4.1.1.2 Macrophages

Macrophages are a heterogeneous population of immune cells that act as regulators of inflammation. TRPA1 mediates inflammation by inhibiting macrophage activation and inducing macrophage apoptosis. TRPA1 is upregulated in atherosclerotic plaques and modulates the transition of macrophages to an anti-inflammatory phenotype, thereby reducing atherosclerosis (Wang et al., 2020). The TRPA1 agonist cinnamaldehyde significantly suppressed the IL-1β expression in phorbol 12-myristate 13-acetate-stimulated macrophages, while TRPA1-KO aggravated macrophage infiltration, renal tubular damage, and renal dysfunction in mice (Ma et al., 2019). Moreover, it has been reported that the TRPA1 agonist cannabinoid reduces INF-γ in macrophages by inhibiting nitric oxide (NO) production to ameliorate colitis in mice (Romano et al., 2013). Therefore, the activation of the TRPA1 channel exerts an anti-inflammatory effect by regulating the release of inflammatory factors from macrophages.

### 4.2 Inflammatory factors and TRPA1

Cannabidiol (CBD), a non-toxic phyto cannabinoid derived from cannabis, has anti-inflammatory effects under a variety of inflammatory conditions. Past studies have reported that, in RA, CBD activates TRPA1 and mitochondrial targets, which reduces the production of IL-6 and MMP-3 in synovial fibroblasts to exert anti-inflammatory effects (Lowin et al., 2020). Carvacryl acetate, a receptor agonist of TRPA1, reduces the release of neutrophils and proinflammatory cytokine IL-1β in the jejunum of irinotecan (CPT-11)-induced mucositis mice, thereby exerting anti-inflammatory and therapeutic effects on bacteremia (Alvarenga et al., 2017).

## 5 Therapeutic applications of TPRA1 agonists in inflammatory diseases

### 5.1 Cardiovascular and cerebrovascular diseases

The pathology of inflammation is related to ischemia-reperfusion injury and is also associated with the occurrence and development of the disease. TRPA1 is widely expressed in vascular cells and regulates cell membrane potential, signal transduction, hemodynamics, vasomotor function, and vascular remodeling. It is also associated with exhibiting a protective role in ischemia-induced myocardial and brain tissues (Aubdool et al., 2016; Delgermurun et al., 2016). The TRPA1 activator ASP7663 attenuated myocardial injury in a rat model of ischemia-reperfusion (IR) and reduced cardiomyocyte death (Bandell et al., 2004; Lu et al., 2016). The TRPA1 agonist allicin possesses vasodilatory effects on the vascular endothelium which protects against coronary endothelial dysfunction (Sun and Ku, 2006). Qian et al. (2013) suggested that the stimulation of TRPA1 extends Ca^2+^ signaling effector coupling along endothelial cells, resulting in cerebral arterial vasodilation. The activation of endothelial TRPA1 induces Ca^2+^ influx and the relaxation of the intracranial vessels in pressurized rats (Earley, 2011). Sullivan has reported that TRPA1 is a sensor of hypoxia-ischemia and that TRPA1 channel deletion in endothelial cells increases inflammation and promotes cerebral infarction. In WT mice, the activation of TRPA1 with cinnamaldehyde reduced the infarct size (Sullivan et al., 2015; Pires and Earley, 2018). Atherosclerosis is a chronic inflammatory disease. In support, Zhao et al. (2016) reported that, in apolipoprotein E (Apoe)-KO mice, a mouse model of atherosclerosis, AITC inhibited the progression of atherosclerosis by activating TRPA1 to reduce inflammation.

### 5.2 Diabetes and obesity

Inflammation is associated with the occurrence and development of diabetes and obesity. Its signaling pathways, upstream reactions, and downstream products have gained much research interest. TRPA1 is involved in diabetes and obesity. AITC—a potent TRPA1 agonist—increases glucose uptake, improves insulin signaling, and mitochondrial function, and reduces the expression of obesity-induced associated inflammation factors TNF-α, and IL-6 (Derbenev and Zsombok, 2016). Similarly, the intake of the TRPA1 agonist cinnamaldehyde reduces visceral adipose tissues and liver damage in mice fed with a high-fat and high-sucrose diet (Tamura et al., 2012). Glucagon-like peptide 1 (GLP-1) inhibits the development of inflammatory diseases (Sartorius et al., 2014). Emery et al. (2015) depicted that TRPA1 exists in mouse intestinal endocrine cells and that its activation promotes GLP-1 in a calcium-dependent manner, which might be a target for the treatment of diabetes.

### 5.3 Kidney injury and sepsis

TRPA1 is well associated with ischemia and reperfusion. In the renal tissues of IR injury, tubular epithelial cells slough off, inflammatory cells infiltrate, and a series of inflammatory responses appear (Wu et al., 2019) demonstrated that TRPA1-KO mice aggravated renal IR injury (Ma and Wang, 2021) Inflammation, microcirculation dysfunction, and metabolic remodeling are the pathogenesis of acute kidney injury in sepsis. In animal studies, TRPA1 prevented sepsis or IR renal injury by downregulating macrophage-mediated inflammation. TRPA1 prevents sepsis-induced organ damage and death. It reduces cecal ligation and puncture (CLP)-induced mitochondrial oxidative stress and enhances mitochondrial homeostasis. Moreover, TRPA1 protects the kidney from sepsis-related injury by promoting mitochondrial biosynthesis and homeostasis. Furthermore, the A-967079 inhibitor of TRPA1 exacerbates inflammatory responses and renal injury (Zhu et al., 2018).

### 5.4 Psoriasis

TRPA1 is a potential drug target for inflammatory skin diseases. The TRP-neuropeptide pathway plays an important role in the pathogenesis of psoriasis (Kodji et al., 2019). TRPA1 is involved in a range of skin physiological functions, including the formation and maintenance of the physicochemical skin barrier as well as the growth, and differentiation of skin cells and tissues (Maglie et al., 2021). The topical application of the TRPA1 activators such as IMQ and TRPA1 causes the activation of dermal γδ T cells *via* IL-23. It also triggers the skin neuroimmune regeneration cascade and promotes tissue regeneration in adult mammals, thereby providing a new avenue for wound and scar treatment research (Wei et al., 2020).

### 5.5 Enteritis

The mediating effect of TRPA1 on the pathogenesis of enteritis remains unclear. According to the literature TRPA1 plays dual effects (pro-inflammatory and anti-inflammatory) in the occurrence and development of enteritis. In the early stage of inflammation, the activation of TRPA1 promoted the release of inflammatory mediators such as SP, which has a pro-inflammatory effect. However, further aggravation induces a change in the function of TRPA1, thereby improving inflammation. The levels of pro-inflammatory mediators SP, NKA, and IL-1β were significantly increased in WT mice when compared with that in TRPA1 KO mice, and the lack of TRPA1 reduced the microbial community in the gut (Kun et al., 2014). Kun *et al.* reported a protective role of TRPA1 activation in the colitis response, while TRPA1 inhibited CD4^+^ T cell activation and the colitis response (Bertin et al., 2017). TRPA1 is expressed in the sensory fibers that innervate the gastrointestinal tract and mediates the hypersensitivity of the gastrointestinal tract to mechanical stimuli. Capsazepine (CPZ) first activates TRPA1, followed by deep and persistent desensitization, resulting in an anti-inflammatory effect. CPZ enema resulted in an anti-inflammatory effect on TRPA1-KO mice (Kistner et al., 2016). Although neuronal TRPA1 increases acute inflammation, non-neuronal TRPA1 activation is anti-inflammatory, and TRPA1 in CD4+T cells reduces T cell-mediated colitis. In addition, cannabidivarin (CBDV) activates and desensitizes TRPA1 and reduces the cytokine expression in colon biopsies from pediatric patients with ulcerative colitis (Pagano et al., 2019). Therefore, TRPA1 plays a bidirectional regulatory role in enteritis, which is dependent on the disease stage.

**FIGURE 1:**
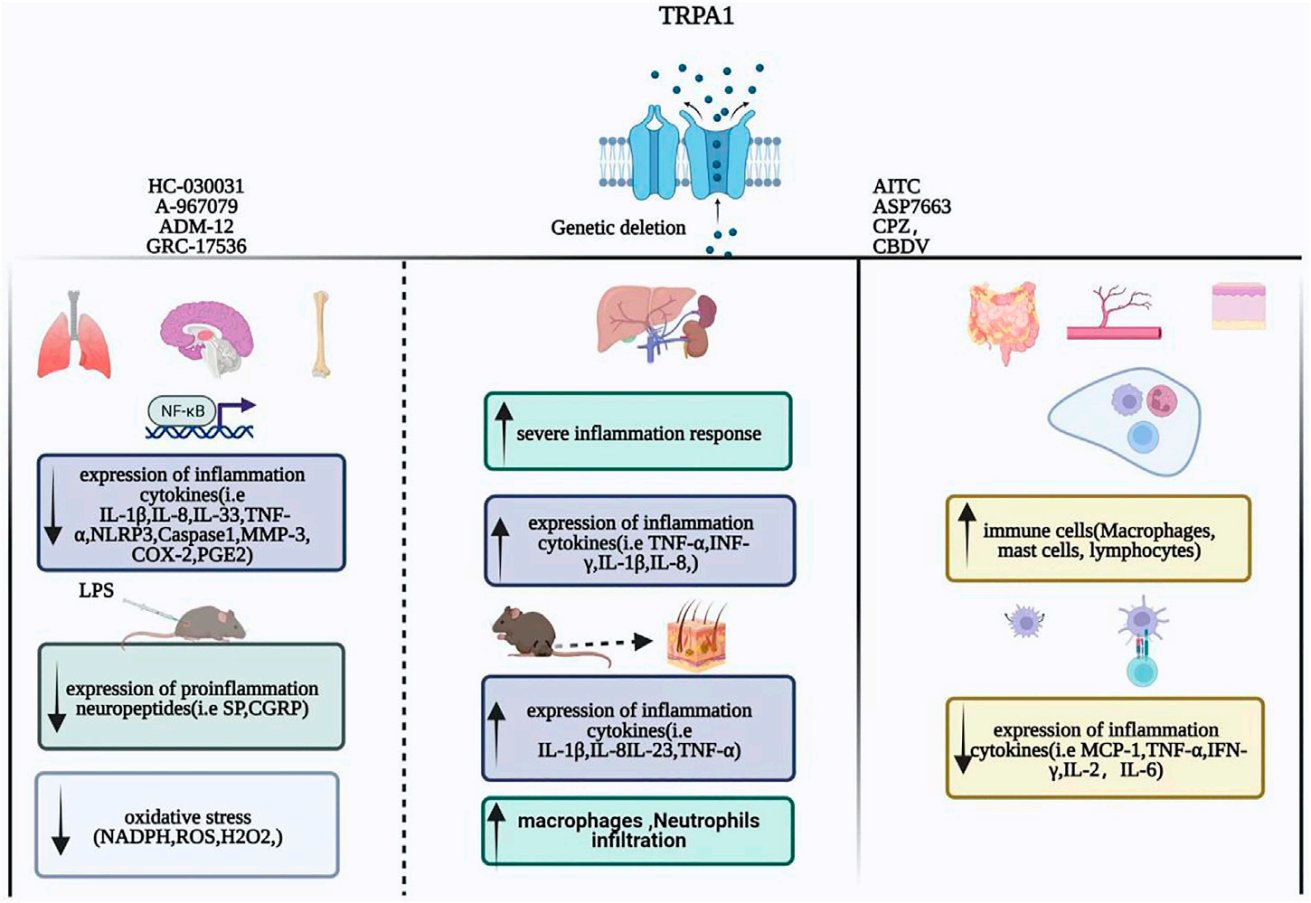
Inflammation: The role of the TRPA1 channel.

In summary, TRPA1 plays an anti-inflammatory role in several inflammatory diseases by regulating T-cell activation, inhibiting the release of inflammatory factors and chemokines from macrophages, and reducing the expression of pro-inflammatory receptors. TRPA1 is activated to treat enteritis, cardiovascular, cerebrovascular inflammatory diseases, and inflammation caused by diabetes, obesity, kidney damage, and sepsis. (Figure 1).

## Conclusion

TRPA1 regulates the inflammatory responses under different disease conditions by regulating inflammatory factors, chemokines, macrophages, and T cells. On one hand, the relationship between TRPA1 and the inflammatory factors IL-1, IL-6, TNF, and IL-8 is related to inflammation-related signaling pathways TLR4/NF-κB and JNK/MAPK. On the other hand, TRPA1 acts as a detector of cellular stress and tissue damage, regulates macrophage polarization, and affects inflammatory factors by promoting the differentiation and activation of T cell expression, thereby playing the protective role of a “sentinel” in the inflammatory response mechanism. In addition, pharmacological antagonists of TRPA1 exert an anti-inflammatory protective effect by blocking the transmission of inflammatory signals, reducing the release of inflammatory factors, and reducing the apoptosis of immune cells. Its agonists exert anti-inflammatory protective effects through regulation of vascular tension and cell membrane potential, which improves mitochondrial synthesis and homeostasis, and through regulation of macrophage-mediated inflammatory responses.

However, the following questions remain unanswered, 1) For instance, although the pro-inflammatory role of TRPA1 is ertremely clear, it remains unclear as to how TRPA1 regulates the intracellular signaling pathways and plays an anti-inflammatory role in inflammatory cytokines and what is the specific mechanism by which TRPA1 plays a different role in the same inflammatory disease. 2) The lack of specific and reliable antibodies against mouse TRPA1 and the lack of knowledge about its specific action pathway, the related upstream and downstream mechanisms, and regulatory mechanisms of the peripheral and central nervous systems and the immune system remain unclear until date. 3) The specific functions of TRPA1 were determined through interactions among proteins, stimulatory factors, and the oxidative state of cells, albeit the specific mechanism warrant verification through further experiments. 4) What are the endogenous ligands of TRPA1? and whether TRPA1 differs in its physiological and pathological functions across species and whether the huge species differences, especially between mice and humans, hinder the translation of preclinical to clinical applications regarding TRPA1 function remain unanswered. 5) Moreover, Long-term use of receptor agonists can lead to desensitization, the optimal treatment indication and the activation mechanism are unclear. 6) Whether there is a cross-effect between TRP channels. Therefore, future studies should characterize the functional properties of TRPA1 in immune cells and nervous cells as an important step toward understanding its role in inflammation and its potential as a therapeutic target. Recent years have witnessed the rapid development of TRPA1 small molecule inhibitors and agonists, and they are expected to be potential drugs useful for the treatment of inflammatory diseases. However, the regulation of the ion channel activity is a complex and dynamic process related to several factors, such as the drug concentration, the mode of administration, inflammatory mediators, and neurological status. Understanding and resolving these issues can reveal the physiological functions of TRPA1 as well as broaden the horizons for developing new therapeutic options in the future.

## Glossary

AD: Alzheimer's disease
AMPK: Activated protein kinase
Apoe: apolipoprotein E
ATP: adenosine triphosphate
Aβ: Amyloid-β
Bcl-2: B-cell lymphoma-2
Ca2+: Calcium
CBD: Cannabidiol
CBDV: cannabidivarin
CEES: 2-chloroethyl ethyl sulfide
CD36: cluster of differentiation 36
CFA: complete Freund’s adjuvant
CGRP: cysteinyl aspartate calcitonin dene-related peptide
ConA: ConcanavalinA
CPZ: Capsazepine
CXCL1: CX-C ligand
CHOP: CCAAT/enhancer-binding protein homologous protein
COX-2: cyclooxygenase-2
CSE: cigarette smoke extract
DINP: di-iso-nonyl phthalate
DNCB: 2,4-dinitrochlorobenzene
Dox: doxorubicin
DRG: dorsal root ganglion
GLP-1: Glucagon-like peptide 1
GPCRs: G protein-coupled receptors
4-HNE: hydroxynonenal
HIF1: hypoxia-inducible factor-1
H2O2: hydrogen peroxide
IBD: inflammatory bowel disease
IFN-γ: interferon-gamma
IL-1: interleukin-1
IL-6: interleukin-6
IL-8: interleukin-8
IMQ: imiquimod
I/R: ischemia and reperfusion
JNK: c-Jun N-terminal kinase
LFN: leflunomide
LECs: lung epithelial cells
MCP-1: monocyte chemotactic protein-1
M-CSF: macrophages colony-stimulating factor
MGO: Methylglyoxal
MSU: monosodium urate
MMP-9: matrix metalloprotein 9
NADPH: nicotinamide adenine dinucleotide phosphate
NAEA: N-acylethanolamines anandamide
NF-kB: nuclear factor kappa-B
NKA: neurokinin A
NLRP3: NOD-like receptor thermal protein domain-associated protein 3
NK1: neurokinin-1
NMDA: N-methyl-D-aspartate
NO: nitric oxide
OA: osteoarthritis
PGE2: prostaglandin
PKC: protein kinase C
PLC: Phospholipase
p38 MAPK: PP2B, protein phosphatase 2B
RA: rheumatoid arthritis
RNS: reactive nitrogen species
ROS: reactive oxygen species
RTKs: receptor tyrosine kinases
TF: transcription factors
ST2: suppression of tumorigenicity 2 receptor
SM: Sulfur mustard
SP: specific proteinase, substance P
Th1: T helper type l
Th2: T-helper type2
Th17: T helper typel7
TLR-2: toll-like receptor 2
TNBS: trinitrobenzene-sulfonic-acid
TNF-α: tumor necrosis factor-α
TRPA1: transient receptor potential ankyrin 1
TRPV1: transient receptor potential vanilloid 1
WT: wide-type
